# Psychiatric manifestations of autoimmune encephalitis

**DOI:** 10.1192/j.eurpsy.2021.1265

**Published:** 2021-08-13

**Authors:** M.T. Valadas, R. Mota Freitas, R. Varela

**Affiliations:** 1 Serviço De Psiquiatria, Unidade Local de Saúde do Baixo Alentejo, Beja, Portugal; 2 Departamento De Psiquiatria E Saúde Mental, Hospital do Espírito Santo de Évora, Évora, Portugal; 3 Serviço De Neurologia, Centro Hospitalar Universitário do Porto, Porto, Portugal

**Keywords:** Autoimmune encephalitis, psychiatric manifestations

## Abstract

**Introduction:**

Autoimmune encephalitis (AE) refers to a newly described, heterogeneous group of rare diseases characterized by brain inflammation and circulating autoantibodies. Various AE have been described and each of them is linked to the presence of specific autoantibodies directed against synaptic and neuronal cell surface antigens. The clinical picture includes a wide array of neuropsychiatric symptoms and is correlated with the associated antibody subtype. Since pronounced psychiatric symptoms are relatively common at the onset, patients can be misdiagnosed and initially driven to psychiatric institutions, thus delaying the adequate diagnosis and management of AE.

**Objectives:**

We aim to review and summarize the psychiatric manifestations of AE that might dominate the clinical picture. We also aim to describe the clinical signs that should alert the psychiatrist to the possibility of these diagnoses.

**Methods:**

We performed an updated review in the PubMed database using the terms “autoimmune”, “encephalitis” and “psychiatric manifestations”. The included articles were selected by title and abstract. We also consulted a reference textbook.

**Results:**

We summarize the reported psychiatric manifestations of AE and also include two situations that can be helpful in AE diagnosis in the psychiatric setting: symptoms that should alert the physician for the possibility of AE and symptoms that should prompt an antibody detection test.

**Conclusions:**

AE are rare diseases that present very frequently with psychiatric symptoms as the first manifestation. Psychiatrists need to be aware of the most common psychiatric manifestations of AE since the early recognition and treatment of AE is fundamental for a good outcome.

